# A Rare Tumor in a Rare Location: Radiology and Pathology Findings With a Literature Review on Intraventricular Gliosarcoma

**DOI:** 10.7759/cureus.34622

**Published:** 2023-02-04

**Authors:** Kavya Mirchia, Mary T Mahoney, Omari Christie, Christine E Fuller, Kanish Mirchia

**Affiliations:** 1 Radiology, State University of New York Upstate Medical University, Syracuse, USA; 2 College of Medicine, State University of New York Upstate Medical University, Syracuse, USA; 3 Pathology, State University of New York Upstate Medical University, Syracuse, USA; 4 Pathology, University of California San Francisco, San Francisco, USA

**Keywords:** mri, gliosarcoma, case report, idh wild type, intraventricular tumor

## Abstract

Gliosarcoma (GS) is an extraordinarily rare variant of glioblastoma, which is differentiated by its distinct biphasic histopathological morphology consisting of both glial and mesenchymal elements. Although GS has a predilection for the cortical hemispheres, rare occurrences of intraventricular gliosarcoma (IVGS) have been documented in the literature. In this report, we present a 68-year-old female patient with a primary IVGS arising from the frontal horn of the left ventricle with corresponding left ventricular entrapment. The clinical course as well as associated tumor features as observed on computed tomography (CT), magnetic resonance imaging (MRI), and immunohistochemical studies are presented along with a relevant review of the current literature.

## Introduction

Gliosarcoma (GS) is an exceedingly rare WHO grade 4 neoplasm, usually classified as an isocitrate dehydrogenase (IDH) wildtype genetic rare variant of glioblastoma (GBM), which comprises about 2% of glioblastomas [1,2]. GS is differentiated from GBM by the distinct biphasic morphology showing both glial and metaplastic mesenchymal components [2-5]. The glial component can be highlighted by immunopositivity for glial fibrillary acidic protein (GFAP), whereas the metaplastic component can show differentiation into fibroblast-like, myogenic, osseous, chondroid, and adipocytic cell lines [1,2]. Despite these morphological differences, GS is clinically analogous to GBM [2], with a similarly abysmal prognosis and a median survival of 16.8 months [3].

GS usually presents as a supratentorial intraparenchymal lesion abutting the meninges, which is not unlike the presentation of a meningioma and has a predilection for the temporal, frontal, parietal, and occipital lobes in order of preference [4-6]. Exceptionally rare occurrences of GS in the intraventricular location have been reported in the literature [4-16]. However, given the lack of discrete imaging characteristics, intraventricular gliosarcoma (IVGS) can often be misdiagnosed as a lower-grade astrocytoma on initial evaluation [6]. Given its high connective tissue content, gliosarcoma also has the gross appearance of a well-circumscribed mass, resembling a meningioma.

In this report, we describe a novel case of a 68-year-old female with primary IDH-wildtype intraventricular gliosarcoma (IVGS) arising from the frontal horn of the left ventricle with resultant left ventricular entrapment, confirmed by histopathological evaluation. A review of the current IVGS literature was conducted, along with a comparison of associated tumor radiologic findings. 

## Case presentation

**Figure 1:**
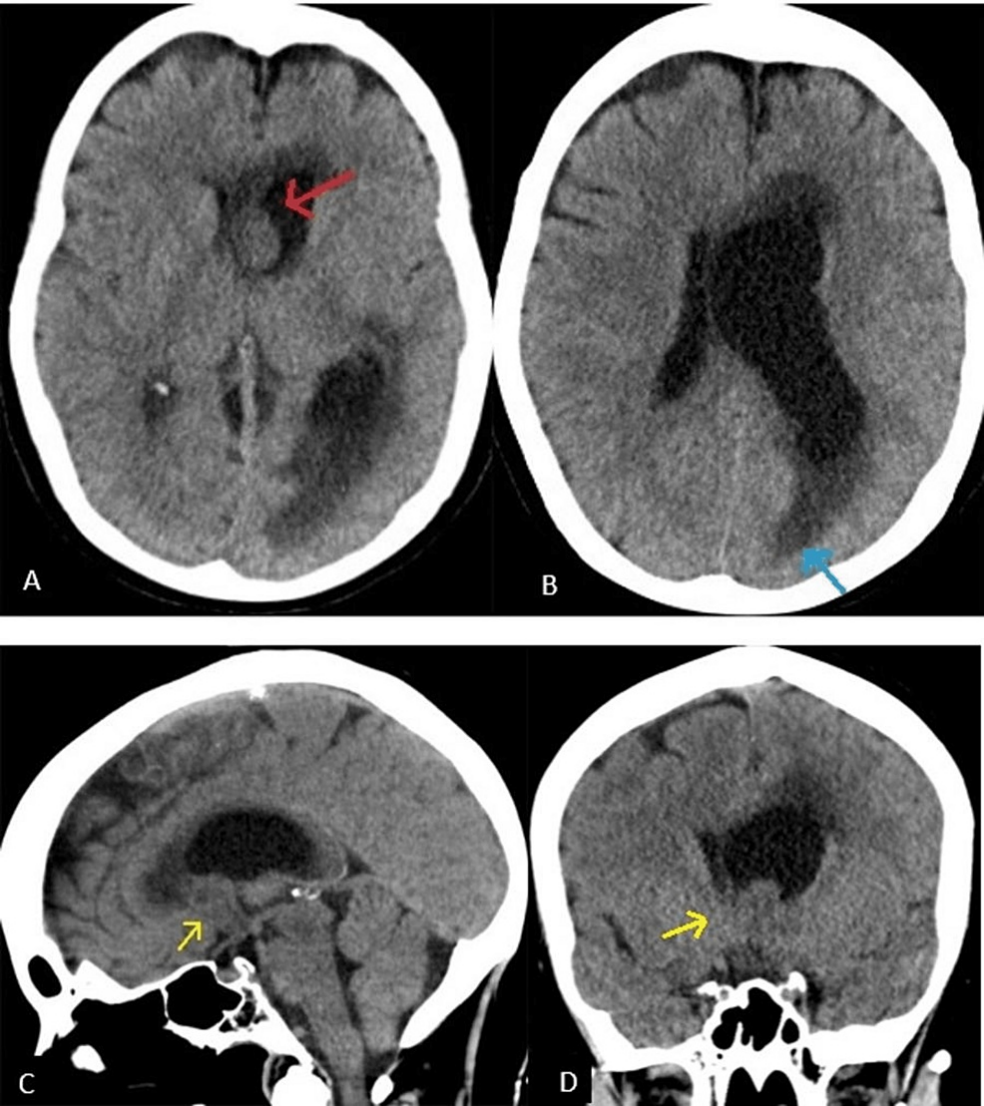
CT findings Axial (a and b), sagittal (c), and coronal (d) non-enhanced CT images of the brain. An intraventricular nodule is demonstrated (red arrow) with asymmetric dilation of the left lateral ventricle. There is a midline shift, most notable at the level of the interventricular septum. Periventricular hypoattenuation (blue arrow) is suggestive of transependymal edema, an effect of acute hydrocephalus. There is an extension of the nodule into the foramen of Monro (yellow arrow).

**Figure 2:**
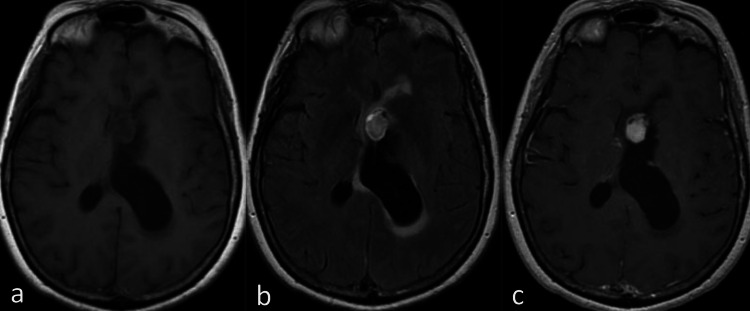
MRI findings Axial T1 w/o (A), T2 Flair (B), and T1 with (C) MR images. The lesion arises at the level of the foramen of Monro and is T1 hypointense and T2 hyperintense. Periventricular T2 hyperintensity and T1 hypointensity indicate transependymal edema, sequelae of ventricular dilatation, and elevated intraventricular pressure. There is heterogeneous enhancement of the lesion.

A 68-year-old woman with no history of prolonged contraception use presented with a one-month history of subjectively reported "fogginess" with now acute and worsening confusion, aphasia, and subtle right-sided weakness over the past two days. The physical exam demonstrated right-sided hemiparesis, right facial droop, and pronator drift. The initial CT scan without contrast demonstrated a 2.1 x 0.7 x 1.3 cm (anterior-posterior (AP) x transverse (TV) x craniocaudal (CC)) heterogeneous left ventricular soft tissue nodule extending into the interventricular foramen, causing left ventricular entrapment, asymmetric dilation of the left lateral ventricle sparing the left frontal horn, and an 8 mm rightward midline shift (Figure 1). Subsequent MRI imaging demonstrated a T1 hypointense, T2 hyperintense, heterogeneously enhancing 1.8 x 1.7 cm intraventricular mass arising from the frontal horn of the left ventricle at the level of the foramen of Monro (Figure 2). MRI evaluation demonstrated periventricular T2 fluid-attenuated inversion recovery (FLAIR) hyperintensity, asymmetric to the left, consistent with transependymal edema indicative of increased intraventricular pressure. Further radiographic evaluation was negative for metastatic disease.

**Figure 3:**
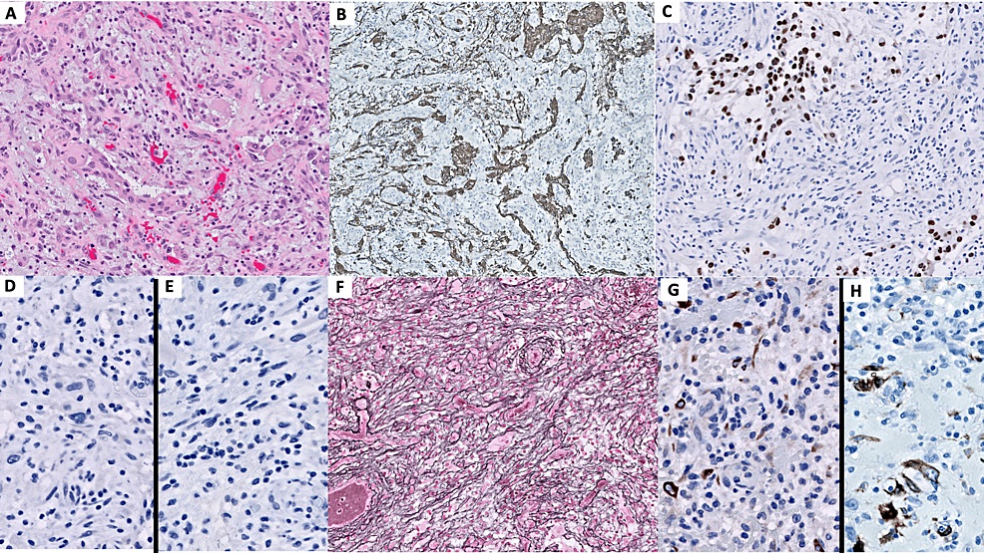
Histological tumor findings The tumor (A: hematoxylin and eosin) shows patchy positivity with glial markers GFAP (B) and OLIG2 (C), immunonegativity for IDH1 R132H (D) and BRAF V600E (E) mutant proteins, extensive deposition of reticulin (F), and focal positivity with muscle markers desmin (G) and smooth muscle actin (SMA) (H).

Within days, the patient underwent subtotal lesion resection and septum pellucidotomy. A gross surgical specimen demonstrated a nodular, circumscribed mass composed of gray-tan tissue. Microscopic examination revealed a highly pleomorphic, mitotically active neoplasm (Figure 3a). The tumor was composed of nests of tumor cells with glial morphology, expressing GFAP (Figure 3b) and OLIG2 (Figure 3c), and admixed sarcomatous elements showing increased pericellular reticulin deposition (Figure 3f) and focal myogenic differentiation (desmin and SMA, Figure 3g and Figure 3h). The isocitrate dehydrogenase 1 (IDH1) R132H, histone 3 genes (H3K27M), enhancer of zeste homolog inhibitory protein (EZHIP), and B-Raf proto-oncogene (BRAF)-V600E immunohistochemical stains were negative (Figure 3d and Figure 3e). α-thalassemia mental retardation X-linked protein (ATRX) immunohistochemistry retained nuclear expression, p53 immunohistochemistry showed strong nuclear positivity in approximately 3% of tumor nuclei, and the Ki-67 labeling index was moderately elevated at approximately 10%.

**Figure 4:**
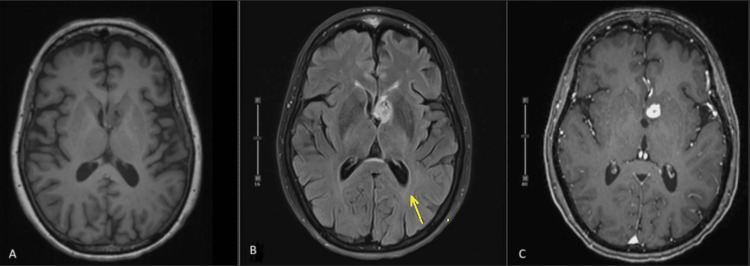
Post-treatment MRI Axial T1 images without contrast. (a) T2 FLARE (b) contrast-enhanced T1 (c) images of the brain post-chemoradiotherapy and subtotal resection. Images demonstrate a residually enhancing lesion along the interventricular septum. The ventricular dilatation and associated transependymal edema have resolved (yellow arrow).

Postoperatively, there was persistent hydrocephalus that was treated with an external ventricular drain. Repeat MRI imaging one month after resection demonstrated a resolved rightward midline shift and an appropriate interval decrease in periventricular FLAIR hyperintensity. There were expected imaging findings of a subtotal resection with postoperative MRI (Figure 4), which demonstrated subtotal resection with the residual enhancement of the inferior margin of the left frontal horn measuring 1.2 x 1.1 x 0.9 mm (TV x AP x CC).

The patient is now four months post-subtotal resection and six weeks into concurrent chemoradiotherapy with temozolomide (TMZ). She reports short-term memory loss without additional neurological symptoms. All of the aforementioned initially presenting neurological symptoms, including hemiparesis and aphasia, have resolved on the most recent follow-up examination. Treatment is ongoing at the time of publication with adjuvant TMZ chemotherapy.

## Discussion

Analogous to glioblastoma, GS occurs most commonly in the fifth to seventh decade of life with a male:female ratio of 1.4:1 [4,5]. IVGS has been reported 14 times in the literature (Table 1). One prior report describes a subependymal location with protrusion into the ventricle [7]. The majority of reported IVGS cases were seen at primary presentation, with two patients having a prior history of ependymoma [8, 17]. To the best of our knowledge, this is the oldest patient with an initial presentation of an IVGS.

**Table 1:** Literature Review of IVGS LV: lateral ventricle; NM: not mentioned. Graphical representation of prior case reports of intraventricular gliosarcoma (IVGS), identifying aspects of; age at diagnosis, tumor type, location within the ventricle, imaging findings, and survival. To date, we currently present the oldest female case of IVGS.

Author	Patient Age (years), Gender (♀/♂)	Tumor location	Imaging findings	Follow-up: Overall survival after initial surgical resection, evidence of neuraxial invasion
Baldawa et al., 2013 [8]	18♀	Temporal horn of the left LV and atrium	CT: iso-to-hyperdense with homogenous enhancement and speckled calcification; MRI: Isotense T1WI, hyperintense T2WI, homogenous enhancement with the enhancement of the ependymal lining.	Four months (alive); drop metastases in the temporal lobe and transependymal spread
Doddamani et al., 2016 [5]	23♂	Occipital horn of the right LV	CT: cystic lesion with hyperdense mural nodule; MRI: hyperintense T1WI, iso- to hyperintense T2WI, heterogeneously enhancing cystic lesion with enhancing mural nodule that involves the septum pellucidum	Six months (alive)
Salunke et al., 2017 [12]	28♂	Bilateral LV with involvement of the contiguous right frontal lobe	CT: hyperdense, heterogeneously enhancing lesion; MRI: hypointense T1WI, hyperintense T2WI with heterogeneous enhancement	NM
Higashino et al., 2001 [17]	29♂	Right LV	CT: non-cystic, isodense, heterogeneously enhancing lesion with calcification; MRI: iso- to hypointense T1WI, hyperintense T2WI, heterogeneous enhancement	79 days (deceased); leptomeningeal carcinomatosis
Tuan et al., 2022 [6]	32♀	Trigon region of right LV	CT: NM; MRI: Hypointense T1WI, Hyperintense T2WI, heterogeneous enhancement with thickening of ependymal membrane	10 days (deceased)
Han et al., 2008 [16]	36♂	Bilateral intraventricular and trunk of the corpus callosum	CT: NM; MRI: non-cystic hypointense T1WI, hyperintense T2WI, heterogeneous enhancement	Eight months (alive)
Hsu et al., 2012 [11]	37♀	Left LV with brain parenchyma extension	CT: iso- to hypodense non-cystic lesion; MRI: hypointense T1WI, hyperintense T2W1 with heterogeneously enhanced	12 months (alive); leptomeningeal carcinomatosis of the pontine surface and C1-3 spinal cord
de Macedo Filho et al., 2020 [13]	44♂	Bilateral LV trigone and corpus callosum	CT: NM; MRI: hypointense T1; hyperintense T2; cystic lesion with heterogeneous enhancement.	45 days (deceased)
Huo et al., 2014 [15]	47♂	Anterior horn and body of the left LV	CT: iso- to hyperintense contrast study, NM: normal contrast study; MRI: hypointense T1WI, iso- to hyperintense T2WI, heterogeneous enhancement with the core of hypointensity	130 months (alive)
Han et al., 2008 [16]	54♀	Left posterior horn of LV	CT: NM; MRI: hypointense to isointense T1WI, hyperintense T2WI, heterogeneously enhancing cystic lesion	One month (deceased)
Govindan et al., 2008 [9]	55♀	Septum and frontal horns of bilateral LV	CT: Iso- to hyperdense lesion, NM of contrast study MRI: Hypointense on T1W, hyperintense T2W, heterogeneous enhancing non-cystic lesion	10 days (deceased)
Sarkar et al., 2013 [14]	60♀	The septal region, extending into the body and the frontal horn of the bilateral LV	CT: NM; MRI: isotense T1WI, hypointense T2WI, heterogeneously enhancing noncystic lesion with central necrosis	NM
Moiyada et al., 2010 [7]	65♂	Right temporal horn and atrium of the LV	CT: NM; MRI: isointense T1WI, hypointense T2WI, heterogeneous enhancement with the enhancement of the ependyma of the temporal horn	Two months (alive); transependymal spread
Poyuran et al., 2017 [10]	68♂	Frontal horn of right LV	CT: NM; MRI: isointense T1WI, hyperintense T2WI, homogeneous enhancement	Two months (deceased)
Our Study	68♀	Frontal horn of the LV	CT: Noncystic, heterogeneously enhancing soft tissue lesion; MRI: hypointense T1W, hyperintense T2W heterogeneously enhancing non-cystic lesion	Four months (alive)

The differential diagnosis for an intraventricular tumor primarily includes low-grade glial or glioneuronal tumors, including neurocytomas, subependymomas, and ependymomas, as well as choroid plexus tumors, although astrocytic tumors may be seen as well [6,15]. Additionally, CT and MRI imaging findings can aid in differentiating gliosarcoma from other high-grade gliomas. CT evidence suggestive of GS is an isoattenuating to hyperattenuating, heterogeneously enhancing solid mass with accompanying hydrocephalus in the absence of calcifications [5,6]. MRI characteristics include isointense to hypotense T1 signal and isointense to hyperintense T2 signal in a well-circumscribed, homogeneously enhancing lesion with uneven post-contrast rim-enhancement [5,6]. Of note, all reported cases of IVGS thus far have been located in the lateral or third ventricle, abutting or involving the septum pellucidum [5]. Our case has congruent imaging findings, with CT demonstrating a heterogeneous soft tissue nodule in the left ventricle extending into the interventricular foramen and subsequent MRI imaging demonstrating a T1 hypointense, T2 hyperintense, heterogeneously enhancing intraventricular mass located in the left frontal horn at the level of the foramen of Monro [6]. A similar presentation of an IVGS rising from the left frontal horn of the left ventricle has been reported previously [Hou et al., 15].

Because of the variability of imaging findings and overlapping gross morphology, a histopathological examination of IVGS is still required for a definitive diagnosis. To be classified as a GS, histopathology must demonstrate biphasic morphology consisting of distinct or admixed glial and metaplastic components [1-5]. Examination of this patient’s tumor demonstrated glial elements that stained positively for GFAP and OLIG2, while the metaplastic elements were negative for GFAP and OLIG2 and showed patchy positivity for SMA and desmin. In recent years, there has been an increased interest in the molecular genetic profiles for GS and their role in determining patient prognosis, including the intraventricular variant. GS has a similar but not identical molecular profile to IDH wild-type GBM, including a lower incidence of EGFR amplification, though it is unclear whether this has any prognostic significance.

According to current NCCN guidelines, the standard treatment for GS is maximal safe resection followed by adjuvant radiotherapy and temozolomide (TMZ) chemotherapy. This is the identical treatment protocol that our patient underwent. Even with treatment, the prognosis for GS remains dismal, with a mean overall survival of 17.3 months for primary GS and 10.2 months for secondary GS [3]. The IVGS variant demonstrated a similarly poor prognosis, with a mean survival of 12.9 months as extrapolated from Table 1. However, there have been reports of primary IVGS patients surviving for at least 130 months [Hou et al., 15]. Currently, our patient is alive four months after subtotal resection and has completed six weeks of concurrent adjuvant chemoradiation, with plans to begin another cycle of adjuvant chemotherapy. 

GS displays an increased propensity for metastasis with an incidence of 11%, compared to GBM with an incidence of 0.2-1.2% [2,18]. Both spinal and systemic metastases, most commonly involving the lung, liver, and lymph nodes [18], as well as skull invasion in lobar gliosarcoma, have been reported. This observation is likely a consequence of the capacity and increased propensity for hematogenous spread exhibited by the sarcomatous component. GS also possesses the capacity for intra-axial neural dissemination to the ventricles, spinal cord, and cranial nerves through two proposed pathways: cerebrospinal fluid seeding or transependymal invasion [19]. The intraventricular location of gliosarcoma possesses a greater potential for transependymal spread, as suggested by Moiyadi et al. [7]. The reported literature on IVGS supports this consequence with four reports of neuraxial dissemination [7,8,11,17]. Currently, our patient has no evidence of extracranial or ependymal involvement; however, continued surveillance MRI imaging of the neuraxis is indicated and will be performed.

## Conclusions

Despite increasing reports of intraventricular gliosarcoma over the past decade, primary IVGS still remains an exceedingly rare entity. We report the novel finding of an IDH-wildtype IVGS in a 68-year-old female patient arising from the frontal horn of the left ventricle with corresponding left ventricular entrapment. Although exceptionally rare, IVGS has been well documented and should be considered in the differential diagnosis of intraventricular tumors. Further investigation of prognostic or predictive genetic markers for IVGS will enhance future patient care.

## References

[1] Louis DN, Ohgaki H, Wiestler OD (2007). The 2007 WHO classification of tumours of the central nervous system. Acta Neuropathol.

[2] Lutterbach J, Guttenberger R, Pagenstecher A (2001). Gliosarcoma: a clinical study. Radiother Oncol.

[3] Amer A, Khose S, Alhasan H (2022). Clinical and survival characteristics of primary and secondary gliosarcoma patients. Clin Neurol Neurosurg.

[4] Han SJ, Yang I, Tihan T, Prados MD, Parsa AT (2010). Primary gliosarcoma: key clinical and pathologic distinctions from glioblastoma with implications as a unique oncologic entity. J Neurooncol.

[5] Doddamani RS, Meena RK, Selvam MM, Venkataramanaa NK, Tophkhane M, Garg SK (2016). Intraventricular gliosarcomas: Literature review and a case description. World Neurosurg.

[6] Tuan HX, Hung ND, Minh ND (2022). Primary intraventricular gliosarcoma on MRI: a challenging diagnosis. Radiol Case Rep.

[7] Moiyadi A, Sridhar E, Jalali R (2010). Intraventricular gliosarcoma: unusual location of an uncommon tumor. J Neurooncol.

[8] Baldawa S, Kasegaonkar P, Vani S, Kelkar G (2013). Primary intraventricular gliosarcoma. Clin Neuropathol.

[9] Govindan A, Bhat DI, Mahadevan A, Chakraborti S, Sampath S, Chandramouli BA, Shankar SK (2009). An unusual case of intraventricular gliosarcoma. Clin Neuropathol.

[10] Poyuran R, Bn N, Reddy YV, Savardekar AR (2017). Intraventricular gliosarcoma with dual sarcomatous differentiation: a unique case. Neuropathology.

[11] Hsu WY, Chang YK, Li CF, Chen JC, Chen CY, Tzeng WS (2012). Unusual location of uncommon tumor: one case report of intraventricular gliosarcoma. J Radiol Sci.

[12] Salunke P, Singh H, Vaiphei K (2017). Lateral ventricular gliosarcoma with attachment to septum pellucidum. Asian J Neurosurg.

[13] de Macedo Filho LJ, Barreto EG, Martins PL, Filho EN, Gerson G, de Albuquerque LA (2020). IDH1-mutant primary intraventricular gliosarcoma: case report and systematic review of a rare location and molecular profile. Surg Neurol Int.

[14] Sarkar H, K S, Ghosh S (2013). Pure intraventricular origin of gliosarcoma - a rare entity. Turk Neurosurg.

[15] Huo Z, Yang D, Shen J (2014). Primary gliosarcoma with long-survival: report of two cases and review of literature. Int J Clin Exp Pathol.

[16] Han L, Zhang X, Qiu S (2008). Magnetic resonance imaging of primary cerebral gliosarcoma: a report of 15 cases. Acta Radiol.

[17] Higashino T, Inamura T, Kawashima M (2001). A lateral ventricular gliosarcoma arising in an ependymoma. Clin Neuropathol.

[18] Ramos R, Morais N, Silva AI, Almeida R (2015). Gliosarcoma with neuroaxis metastases. BMJ Case Rep.

[19] Beaumont TL, Kupsky WJ, Barger GR, Sloan AE (2007). Gliosarcoma with multiple extracranial metastases: case report and review of the literature. J Neurooncol.

